# Quantum Dot Infrared Photodetectors: Photoresponse Enhancement Due to Potential Barriers

**DOI:** 10.1007/s11671-010-9767-y

**Published:** 2010-08-31

**Authors:** Vladimir Mitin, Andrei Antipov, Andrei Sergeev, Nizami Vagidov, David Eason, Gottfried Strasser

**Affiliations:** 1 University at Buffalo, SUNY, 332 Bonner Hall, Buffalo, NY 14260-1920, USA

**Keywords:** Quantum dots, Infrared detectors, Photoresponse, Doping, Potential barriers, Capture processes

## Abstract

Potential barriers around quantum dots (QDs) play a key role in kinetics of photoelectrons. These barriers are always created, when electrons from dopants outside QDs fill the dots. Potential barriers suppress the capture processes of photoelectrons and increase the photoresponse. To directly investigate the effect of potential barriers on photoelectron kinetics, we fabricated several QD structures with different positions of dopants and various levels of doping. The potential barriers as a function of doping and dopant positions have been determined using nextnano^3^ software. We experimentally investigated the photoresponse to IR radiation as a function of the radiation frequency and voltage bias. We also measured the dark current in these QD structures. Our investigations show that the photoresponse increases ~30 times as the height of potential barriers changes from 30 to 130 meV.

## Introduction

Many optoelectronic devices are based on the phenomenon of photoconductivity in which a material becomes more electrically conductive due to photocarriers created by electromagnetic radiation. Photocarriers contribute to the electric current until they are trapped by impurities and/or defects. Long photocarrier lifetime would substantially improve the operation of optoelectronic devices, such as IR and THz detectors and solar cells. New nanostructured materials that provide long photocarrier lifetime at room temperatures would significantly increase the commercial market for infrared and terahertz technologies.

Initial hopes related to QD nanostructures were associated with the "phonon bottleneck" concept, which assumes that the phonon-assisted bound-to-bound transitions in QDs are prohibited, unless the energy between two discrete levels matches the phonon energy [1]. According to this concept, the intrinsic electron relaxation in quasi-1D nano-objects, such as QDs, was anticipated to be significantly slower than in 2D and 3D structures. However, the phonon bottleneck model completely ignores interaction between electrons and corresponding modification of electron states. It is not surprising that the experimentally measured phonon-mediated electron relaxation turned out to be much faster than it is expected in the phonon bottleneck concept [2]. Recent investigations [3] unambiguously demonstrated that the actual intradot kinetics is completely opposite to what can be expected for weakly interacting electrons and phonons. In reality, strong coupling between electrons and longitudinal optical (LO) phonons leads to the formation of the polaron states, which decay due to the interaction of LO phonons with acoustical phonons. Such kinetics results in strong energy and temperature dependences of the electron relaxation. For example, for 14 meV transition, the relaxation time reduced from 1.5 ns at 10 K to 560 ps at 30 K, and further to 260 ps at 50 K. At room temperatures, the polaron decay time is observed in the range of 2–30 ps, depending on the electron energy [3]. Thus, after numerous experiments with various QD structures, no true phonon bottleneck has been found [3-5].

Another possibility to suppress photoelectron relaxation and to increase a photoelectron lifetime is related with the interdot kinetics. In theoretical works [6-9], we proposed to suppress the capture processes by means of potential barriers in specially engineered QD structures. Potential barriers are always created, when electrons populating the dots are taken from the specific areas located relatively far from the dots. Changing the position of dopants and doping level, one can manage the potential barriers around dots and control the photoelectron capture processes [8,9].

In this work we present direct experimental demonstration of strong effects of potential barriers on photoresponse of QD structures. The paper is organized in the following way. In the next section we describe the fabrication of the QD structures with different positions of dopants and various doping levels. After that we present results of measurements of the photoresponse and noise characteristics of these structures. Then, using the nextnano^3^ software we calculate the dot population and potential barriers around dots as a function of dopant positions and doping levels. Finally, we discuss the photoresponse enhancement in terms of potential barriers around QDs.

## Device Fabrication

Several standard quantum dot infrared photodetector (QDIP) structures have been grown by the Riber Compact 21 MBE (molecular beam epitaxy) in AlGaAs matrix materials. For a given amount of In, adding aluminum to the nucleation layer and the surrounding matrix decreases the surface mobility of In and, thus, increases the dot density and decreases the dot size, when compared to InAs QDs grown onto GaAs surfaces and embedded in the binary compound GaAs only. In addition to the dot size and density distribution, an increase in the aluminum concentration also increases the conduction band offset. This shifts the center energy (decreases the wavelength) of the detector from the THz to the MID-IR region. In addition, the dark current is further reduced by increasing the aluminum concentration of the matrix material.

Growth temperatures on the substrate surface are monitored by infrared pyrometer. The surface temperature affects the density, quality, and size of the quantum dots that are formed and has to be constant, reproducible, and adjustable to control the QD formation. Typical growth temperatures are 500 ± 10°C as read by the pyrometer.

**Figure 1:**
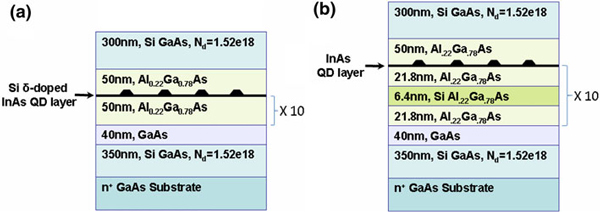
**QDIP structures with *n*-type intra-QD layer doping (a) and inter-QD layer doping (b)**.

The doping for the active layers of the QDIPs can be done in several manners (see Table 1). The QDIP structures with two types of doping have been grown: intra-QD layer doping and inter-QD layer doping. Samples with intra-QD layer doping B44 and B52 have been grown with Si doping in the InAs dots (Figure 1a). The equivalent doping sheet concentration is 2.7 and 5.4 × 10^11^ cm^-2^ for B44 and B52, respectively. Samples with inter-QD layer doping B45, B53, and B54 have been grown with the Si doping directly in the middle of each AlGaAs barrier layer (Figure 1b). The thickness of doped layer is 6.4 nm. The doping sheet concentration is 2.7, 5.4, and 8.1 × 10^11^ cm^-2^ for B45, B53, and B54, respectively. Also, samples with inter-QD layer doping, B46, have been grown with the modulation doping. Each Si-doped 6.4-nm thick layer moves down 2.144 nm in AlGaAs barrier layer. Each repetition ends with 2.5, 4.644, 6.788, 8.932, 11.076, 13.22, 15.364, 17.508, 19.652, and 21.8 nm below AlGaAs barrier layer. The doping sheet concentration is 2.7 × 10^11^cm^-2^.

**Table 1:** Devices

Device	Dopant position	Dopant concentration (×10^11^ cm^-2^)	Number of electrons per QD	Barrier height (meV)
B44	QD layer	2.7	2.4	25
B45	Middle of AlGaAs layers	2.7	2.8	70
B46	Modulation dopping	2.7	2.8	60
B52	QD layer	5.4	4.7	79
B53	Middle of AlGaAs layers	5.4	6.1	130
B54	Middle of AlGaAs layers	8.1	9	200

InAs dots grown on GaAs and AlGaAs surfaces form in approximately 2.2 monolayers of InAs growth. During the normal growth of layers, the substrate is rotated at 30 RPMs to insure uniform thickness of layers.

**Figure 2:**
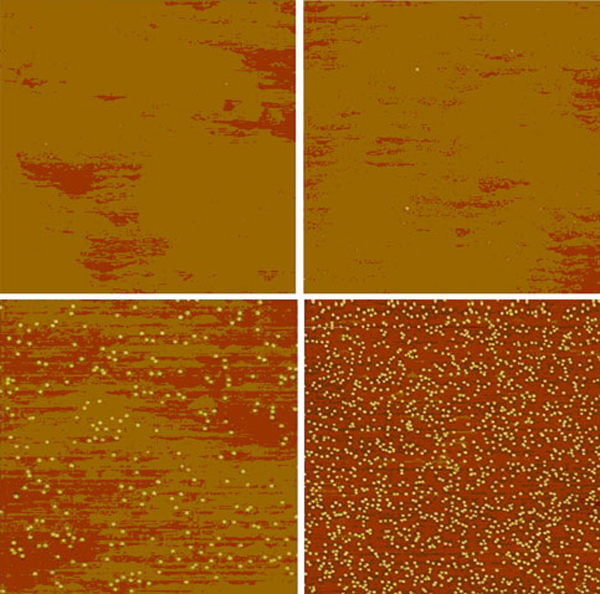
**AFM images of a typical surface quantum dot sample; different positions on a 2 in. wafer; the deposited In increases from *left* to *right* and from *top* to *bottom* going from just a wetting layer (*left upper image*) to a concentration of 4 × 10^10^ dots/cm^2^ in the *right lower image***.

Typically 10 layers of quantum dots are grown in the active layer of the structure. Also, a layer of InAs dots is grown on the final top surface. All the QDIP structures are grown on *n*–type doped GaAs epi-ready substrates. Room-temperature images of surface quantum dots taken at ambient conditions by AFM measurements have been used to calibrate and control the quantum dot size and density. A typical AFM result for InAs quantum dots grown on a GaAs surface is shown in Figure 2: the substrate rotation is stopped during the growth of the QDs to get a density distribution over the 2 in. (or 3 in.) wafer. This gives one side of the wafer, closest to the indium source, a higher density of dots and the other side of the wafer, away from the indium source, a lower density of dots.

The shown images are taken at different positions on a 3-inch wafer with respect to the indium effusion cell. The image size is 3 × 3 μm per image; the distance between images is half an inch, moving closer to the indium cell by going from left to right and from top to the bottom. Further adjustments to growth rates and times allow the properties of the quantum dots to be adjusted. The size of the dots and the matrix material used to embed the QDs determine the wavelength at which the QDIPs operate.

The vertical QDIP structure was processed by standard optical lithography, etching, and metallization techniques. To investigate the electron transport in QDIP structures, square 100 × 100 μm^2^ mesas with alloyed Ni/Ge/Au/Ni/Au high-quality ohmic contacts were formed. Top and bottom contacts were deposited on the highly Si-doped GaAs layers and followed by rapid thermal annealing at 430°C for 40 s. Positive bias polarity corresponds to a positive voltage, which is applied to the top contacts.

## Photoresponse and Dark Current

For low-temperature optical measurements, each sample was mounted inside a helium continuous-flow cryostat. The current–voltage characteristics were recorded with Keithley 2602 Multimeter. Our measurements have been done with 10^-7^ A/cm^2^ accuracy. The spectral response of our QDIP structures was measured using a Bruker Optics Vertex 70 Fourier transform infrared (FTIR) spectrometer and a low noise current mode 7265 DSP Lock-in Amplifier.

**Figure 3:**
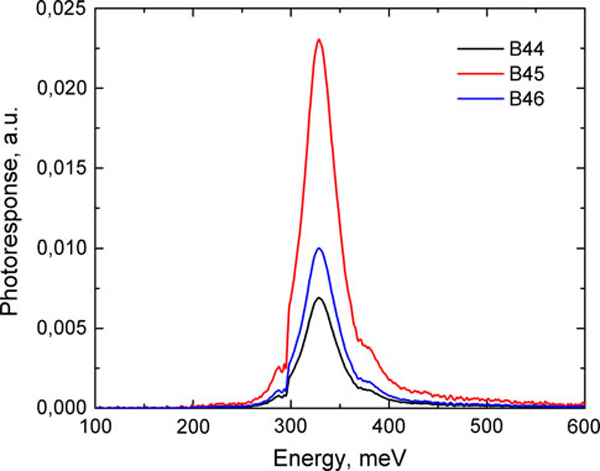
**Normal incidence spectral photoresponse of samples B44–B46 at *T* = 80 K and bias voltage -5 V**.

Normal incidence photoresponse spectra of the B44, B45, and B46 structures at *T* = 80 K and bias voltage -5 V are presented in Figure 3. We observed the maximum response at ~329 meV. Full width at half maximum is ~40 meV. Also, there is an additional small spike that can be clearly observed at the high-energy tail of the spectra. The energy corresponding to the spike is ~380 meV. The valley on the low-energy tail of the spectra at 300 meV corresponds to absorption of IR light by CO_2_ gas in the air. As seen from Figure 3, the photoresponse of B45 is several times larger then that for samples B44 and B46.

**Figure 4:**
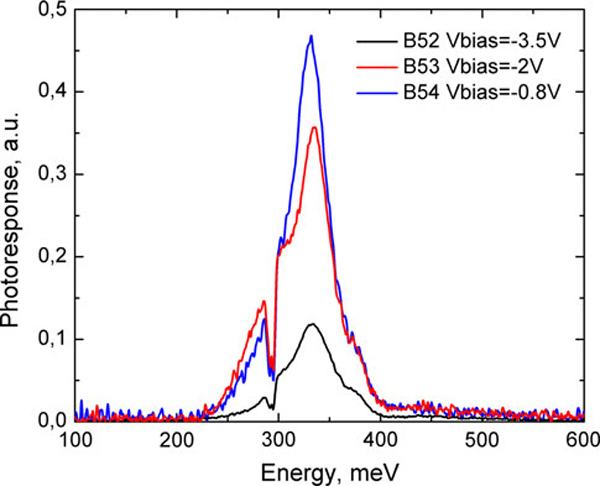
**Normal incidence maximum spectral photoresponse of samples B52–B54 at *T* = 80 K**.

Photoresponse spectra of the samples B52, B53, and B54 are shown in Figure 4. Spectra of all these samples have the same position of a maximum at ~335 meV at negative bias voltages 3.2 V for B52, 2 V for B53, and 0.8 V for B54, correspondingly. The photoresponse of B54 is several times bigger than that of B52. Each spectrum exhibits also local maximums on the low-energy tail at ~300 meV and at the high-energy tail at ~380 meV. Full width at half maximum is ~45 meV. Let us note that at low voltages the photoresponse increases exponentially as well as the dark current density (see next paragraph). At high voltages, photoresponse sharply decreases. The same shapes and positions of maximum in the spectra provide strong evidence that QDs in all our structures are nearly identical.

**Figure 5:**
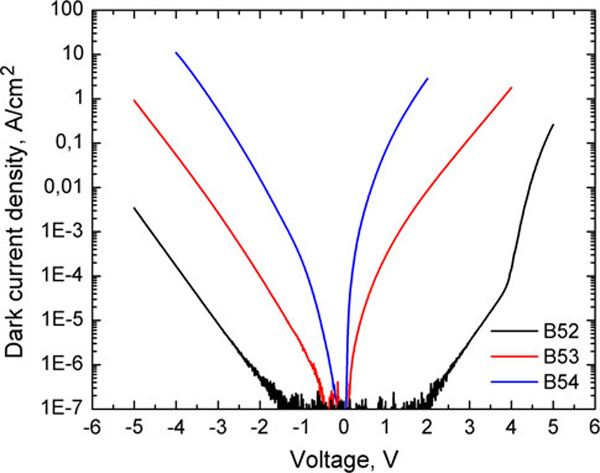
**Dark current density of samples B52–B54 at *T* = 80 K**.

Dark current densities for samples B52, B53, and B54 are presented in Figure 5. The dark current density in the sample B54 is higher by two orders of magnitude than that in the sample B53 and by four orders of magnitude higher than that in B52. Let us note that the current–voltage characteristic for the sample B52 is more symmetrical than that for samples B53 and B54. The asymmetric characteristic of this sample is due to 40 nm GaAs undoped layer at the substrate side.

We also found that the dark current density for sample B46 is higher by two orders of magnitude than that for samples B44 and B45. The sample B46 with the modulation doping has the highest dark current density and the lowest gain. The sample B44 with the silicon Delta doping just before InAs QD layer and the sample B45 with Si doping directly in the middle of each AlGaAs barrier layer show almost the same values of the dark currents.

We investigated the photocurrent in our samples as a function of optical pumping. In these measurements, we used a red light-emitting diode (LED) as a source of the optical radiation. The corresponding energy of photons, ~2 eV, is higher than QDIP's intersubband transitions and InAs energy gap. The energy of IR photoexcitations was tuned to the resonance absorption, i.e. to 380 meV in accordance with the data in Figure 3. The optical power of LED was calibrated by using a silicon power sensor.

**Figure 6:**
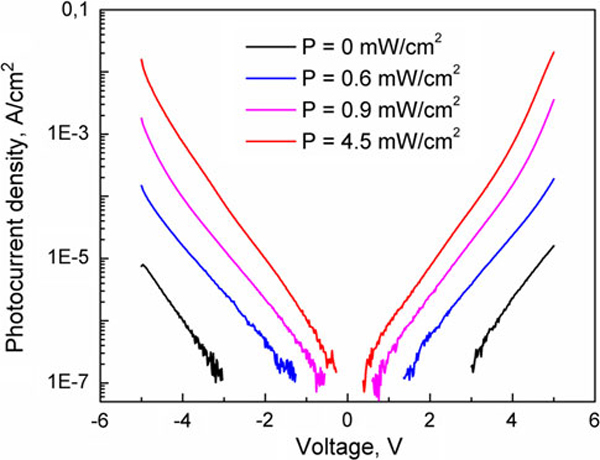
**Photocurrent density of the sample B45 at *T* = 80 K as a function of optical pumping from LED**.

Photocurrent densities for sample B45 at different power of LED's radiation are shown in Figure 6. A photocurrent response increases linearly with a background power and finally saturates at high power. The optical pumping allowed us to increase the photoresponse by three orders of magnitude. This effect is mainly due to an increase in the electron population in the quantum dot energy levels.

## Modeling: Potential Barriers in QD Structures

The analysis of optical and electrical properties of the grown samples was done using nextnano^3^ software [10]. This versatile software allows for simulation of multilayer structures combined of different materials with realistic geometries in three dimensions. The simulation tool solves self-consistently Schrödinger, Poisson, and current equations. The conduction and valence bands are defined within single-band or multiband **k.p** envelope. In this modeling, we used well-established material parameters for the simulated structures: the effective mass of electron, m*, in γ-valley of GaAs is 0.067 and in Al_0.22_Ga_0.78_As, m*, is 0.085. Effects of strain [11,12] were included in simulations.

Using this software, the three-dimensional bandstructures of the grown samples were obtained. Analyzing two-dimensional slices of conduction band profiles, the heights of potential barriers that divide neighboring QDs were defined.

**Figure 7:**
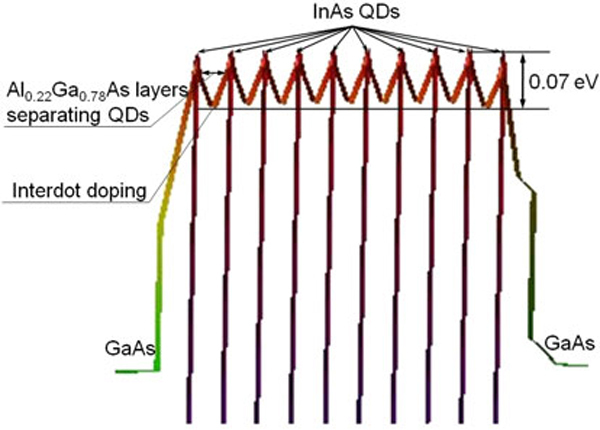
**Two-dimensional slice of the calculated using nextnano^3^ software conduction band structure of B45 sample**.

The height of potential barriers for electrons located in interdot area is defined as the difference between the maximum of the conduction band in QDs and minimum in the depletion regions that occur as a result of interdot doping, as it is shown in Figure 7.

**Figure 8:**
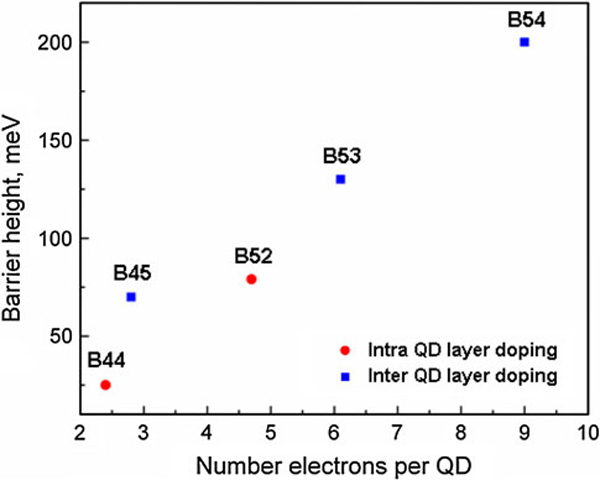
**Barrier height versus number electrons per QD of samples B44, B45, B52, B53, and B54 at *T* = 80 K**.

The calculated heights of these potential barriers at *T* = 80 K in B44, B45, B52, B53, and B54 samples are shown in Figure 8. Point B45 in Figure 8 corresponds to the potential barrier height of 0.07 eV shown in Figure 7.

## Conclusions

In this work we investigated the effects of the potential barriers around QDs on the photoresponse of QDIPs. We found that in accordance with our theoretical conclusions [6-9], the potential barriers substantially suppress photoelectron capture and enhance the photoresponse.

**Figure 9:**
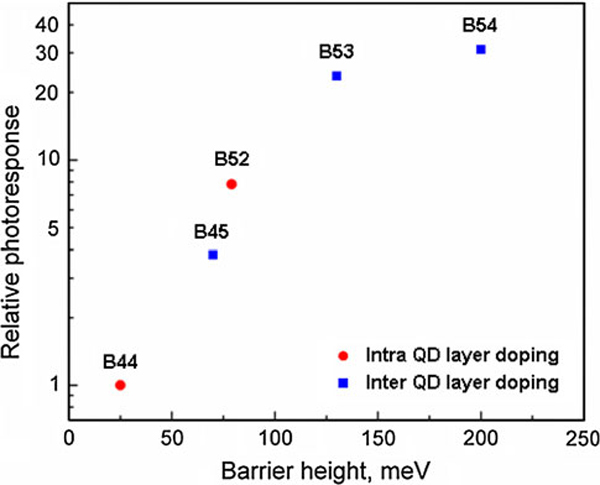
**Normal incidence relative spectral photoresponse versus barrier height of samples B44, B45, B52, B53, and B54 at *T* = 80 K**.

In Figure 9 we summarize our main results. As seen, when the height of the potential barriers in the selected devices changes from 28 to 130 meV, the photoresponse increases exponentially as a function of the barrier height [6-9]. At high doping level (the sample B54), the effect is saturated. We observed ~30 times enhancement of the photoresponse due to potential barriers around dots. Our results show that QDIPs can substantially outperform quantum-well photodetectors due to the manageable photocarrier kinetics, which can be controlled by potential barriers.
